# Rat tail models for the assessment of injectable nucleus pulposus regeneration strategies

**DOI:** 10.1002/jsp2.1216

**Published:** 2022-07-11

**Authors:** Marcos N. Barcellona, Emily E. McDonnell, Shani Samuel, Conor T. Buckley

**Affiliations:** ^1^ Trinity Centre for Biomedical Engineering, Trinity Biomedical Sciences Institute, Trinity College Dublin The University of Dublin Dublin Ireland; ^2^ Discipline of Mechanical, Manufacturing and Biomedical Engineering, School of Engineering, Trinity College Dublin The University of Dublin Dublin Ireland; ^3^ Advanced Materials and Bioengineering Research (AMBER) Centre, Royal College of Surgeons in Ireland & Trinity College Dublin The University of Dublin Dublin Ireland; ^4^ Tissue Engineering Research Group, Department of Anatomy and Regenerative Medicine Royal College of Surgeons in Ireland Dublin 2 Ireland

**Keywords:** degeneration, intervertebral disc, nucleus pulposus, puncture, rat tail model, spine

## Abstract

Back pain is a global epidemiological and socioeconomic problem often associated with intervertebral disc degeneration; a condition believed to initiate in the nucleus pulposus (NP). There is considerable interest in developing early therapeutic interventions to target the NP and halt degeneration. Rat caudal models of disc degeneration have demonstrated significant utility in the study of disease progression and its impact on tissue structure, composition, and mechanical performance. One significant advantage of the caudal model is the ease of access and high throughput nature. However, considerable variability exists across the literature in terms of experimental setup and parameters. The objective of this article is to aid researchers in the design and development of caudal puncture models by providing details and insight into the most reported experimental parameters. Preferred Reporting Items for Systematic Reviews and Meta‐Analyses (PRISMA) guidelines were employed to screen the existing literature and 80 manuscripts met the inclusion criteria. Disc geometry, surgical approaches, effect of needle gauge size to induce degeneration, therapeutic volume, outcome measures, and associated limitations are considered and discussed, and a range of recommendations based on different research questions are presented.

## Graphical abstract

A systematic review on the most reported experimental parameters of rat tail models. Providing a guide to researchers in the design and development of puncture models based on different research questions.

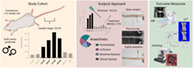

## 1 INTRODUCTION

Intervertebral disc (IVD) degeneration is an etiologically complex condition which remains one of the major contributors of disability at the global scale. The IVD is composed of three distinct compartments: the nucleus pulposus (NP), annulus fibrosus (AF), and cartilaginous endplates (CEPs). The AF is the outermost tissue responsible for resisting torsional stresses and providing structural stability to the disc.^1^ It is largely composed of fibrillar collagens, which are aligned in concentric lamella oriented at approximately 120 degrees from their adjacent layers.^2^, ^3^, ^4^ The CEPs border the disc on upper and lower planes and are composed of a thin layer of hyaline cartilage. The CEP is penetrated by a small vascular network which facilitates nutrient transport and gas exchange into and out of the disc.^5^, ^6^, ^7^ The NP is the innermost compartment and is mostly responsible for the radial distribution of compressive stresses.^8^, ^9^ When healthy, this tissue is a highly hydrated amorphous gelatinous matrix rich in proteoglycans and glycosaminoglycans, containing both fibrillar and nonfibrillar collagens, and smaller quantities of other proteins such as fibronectin and laminins.^8^, ^10^, ^11^, ^12^ While changes have been observed throughout the whole motion segment as degeneration progresses,^13^ degeneration is often believed to initiate within the NP, impairing the biomechanical function of the IVD. As a result, there is considerable research interest in early therapeutic interventions to target the NP and halt degeneration progressing outward through the AF.

Over the years a range of models have been developed to study the origins of disc degeneration, to further elucidate disease progression and to test innovative therapeutic strategies. When developing an in vivo animal model, multiple factors may affect the experimental outcome and need to be taken into careful consideration. Some of these factors include spine anatomy (shape/size), biomechanics as well as biochemical structure and species‐specific pathophysiology.^14^ During aging, the human IVD undergoes the rapid loss of notochordal cells. A similar phenomenon has been observed in goats, sheep, and chondrodystrophic dogs. However, pigs, rabbits, rats, and mice have been reported to retain a notochordal cell population into adulthood.^14^, ^15^, ^16^ Furthermore, different species may be preferred or more suitable depending on the scientific question or objective of the study. For example, genetic models in mice provide biologically relevant insight into factors that lead to degeneration such as sonic hedgehog signaling molecule knockout models, or aging‐associated complications such as collagen and proteoglycan deficiencies.^17^, ^18^ Alternatively, large animal models may be preferred for the clinical translation of regenerative therapeutics and implantable devices in discs of a more anatomically relevant scale comparable to the human IVD.^19^, ^20^

Rodent models, including both mouse and rat, offer significant advantages with regards to high throughput and ease of technology application.^15^ While mouse models offer a wider range of genetic applications, rat models are often preferred when the objective is to deliver treatments locally into the intradiscal space. Within the scope of rat models, tail or caudal discs are of particular interest as they offer considerable advantages in terms of accessibility for surgical procedures, especially when repeated procedures are needed as is the case for surgically induced disc damage and treatment. The objective of this article is to aid researchers in the experimental setup and development of rat tail puncture models by providing details and insight into the most reported experimental parameters.

## 2 IDENTIFICATION OF PEER REVIEWED MANUSCRIPTS

**FIGURE 1:**
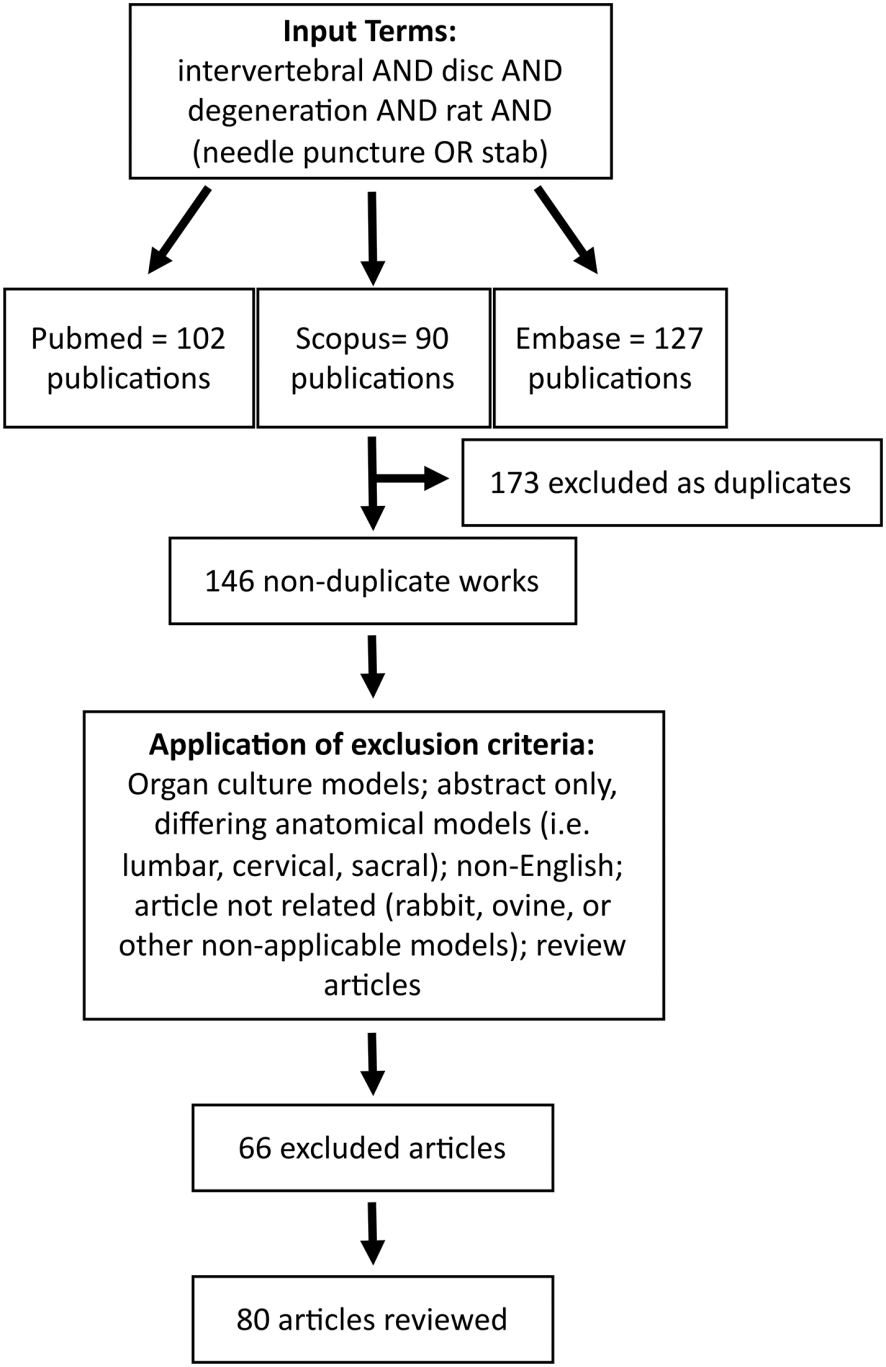
Preferred Reporting Items for Systematic Reviews and Meta‐Analysis (PRISMA) diagram indicating the literature search terms, screening process, and exclusion criteria. One hundred and forty‐six nonduplicate manuscripts were identified across PubMed, Scopus, and Embase on March 7, 2022. Following screening and the application of exclusion criteria, 80 articles were included in this review

Identification of the literature was conducted (initially in August 2021 and updated in March 2022) using the Preferred Reporting Items for Systematic Reviews and Meta‐Analysis (PRISMA) guidelines set forth by Moher et al.^21^ This comprehensive search was conducted in the databases PubMed, Scopus, and Medline using the search terms “intervertebral” AND “disc” AND “degeneration” AND “rat” AND (“needle puncture” OR “stab”). As highlighted in Figure 1, this resulted in 146 unique manuscripts. The exclusion criteria included studies that were conducted using organ culture models, differing anatomical models (i.e., lumbar, cervical, or sacral discs), conference abstracts, articles in non‐English languages, review papers, and nonrelated outputs (i.e., sand rat models or other nonrelevant animal models). After applying these exclusion criteria, a total of 80 manuscripts were retained for review (Table S1).

## 3 SUMMARY OF DATA FROM PEER REVIEWED MANUSCRIPTS

**FIGURE 2:**
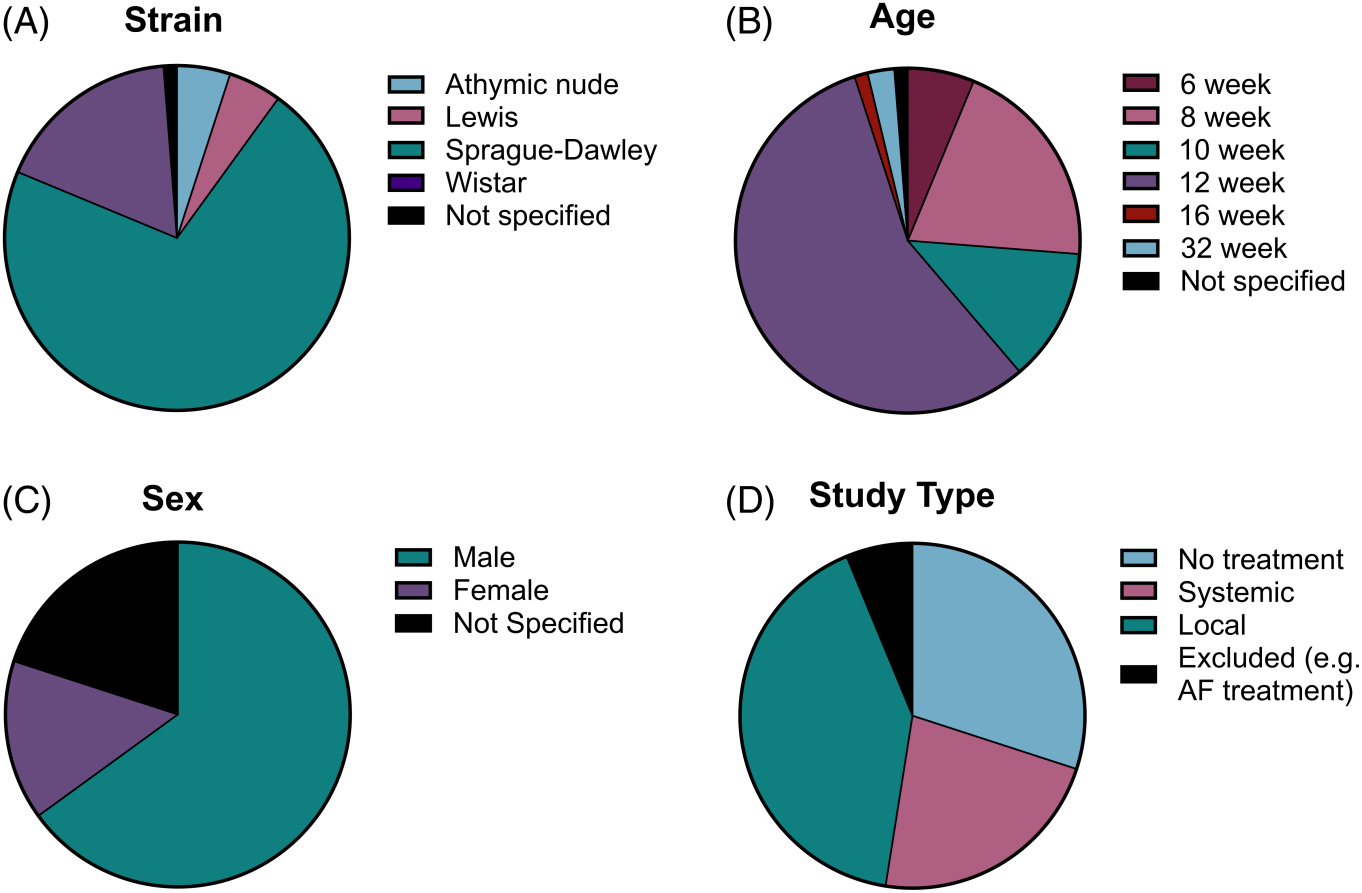
Summary of rat cohort, and the overall study design across the 80 articles reviewed. (A) The frequency of different rat strains used to create degeneration rat tail models (Sprague Dawley = 71.3%, Wister = 17.5%, Lewis = 5.0%, Athymic nude = 5.0% and 1.2% of studies did not specify rat strain). (B) Five studies (6.2%) used periadolescent rats at 6 weeks, while 26 studies (32.5%) used young adult rats at 8 and 10 weeks. However, most studies used rats older than 12 weeks, the timepoint at which skeletally maturity has occurred (12 weeks = 56.3%, 16 weeks = 1.3%, and 32 weeks = 2.5%). (C) Sixty‐five percent of studies used male rats, while only 12.0% of studies used female rats. However, 20.0% of the studies did not specify the sex of the animals used. (D) 41.3% of studies investigated a treatment delivered locally within the degenerated disc, 22.5% focused on systemic delivery such as incorporation of drugs into food, or intraperitoneal injections, while 30.0% solely developed a degeneration model with no treatment delivery

As shown in Figure 2A, almost 90% of all papers reviewed used Wistar or Sprague Dawley rats for their studies (57/80 = 71.3% Sprague Dawley, 14/80 = 17.5% Wistar). Only four studies utilized Lewis rats (5.0%), while one study did not specify the rat strain. Four studies used nude rats (5.0%), which may be preferable or necessary for studies exploring delivery of agents associated with an increased risk of immune responses such as exogenous cell types. There appears to be significant consensus in terms of the age of the rats at the time of the study, with 12‐week animals being used in 45 of 80 (56.3%) of all studies (Figure 2B). Adulthood begins at ~8 weeks of postnatal life and 12‐week‐old rats are widely considered to be skeletally mature animals, while still retaining much of the juvenile phenotype.^22^, ^23^, ^24^, ^25^ This makes them good candidates for studies looking at disease progression versus aging, trauma, or other factors. There was also a considerably higher use of male rats (52/80 = 65.0%) than female rats (12/80 = 12.0%) (Figure 2C). This may be due to the size difference between males and females, which may make both handling as well as intradiscal delivery procedurally easier. However, this bias could have negative implications as recent studies have found that sex differences exist both in disc degeneration as well as in the healing response following injury.^26^ Although it is important to note that 16 studies (20%) did not specify the sex of the rats and these studies may account for additional single sex studies or mixed studies or both. Of the papers reviewed, 33 of 80 (41.3%) provided a local intradiscal treatment, 18 of 80 (22.5%) used systemic treatments and 24 of 80 (30.0%) involved developing a degeneration model and thus provided no treatment (Figure 2D).

**FIGURE 3:**
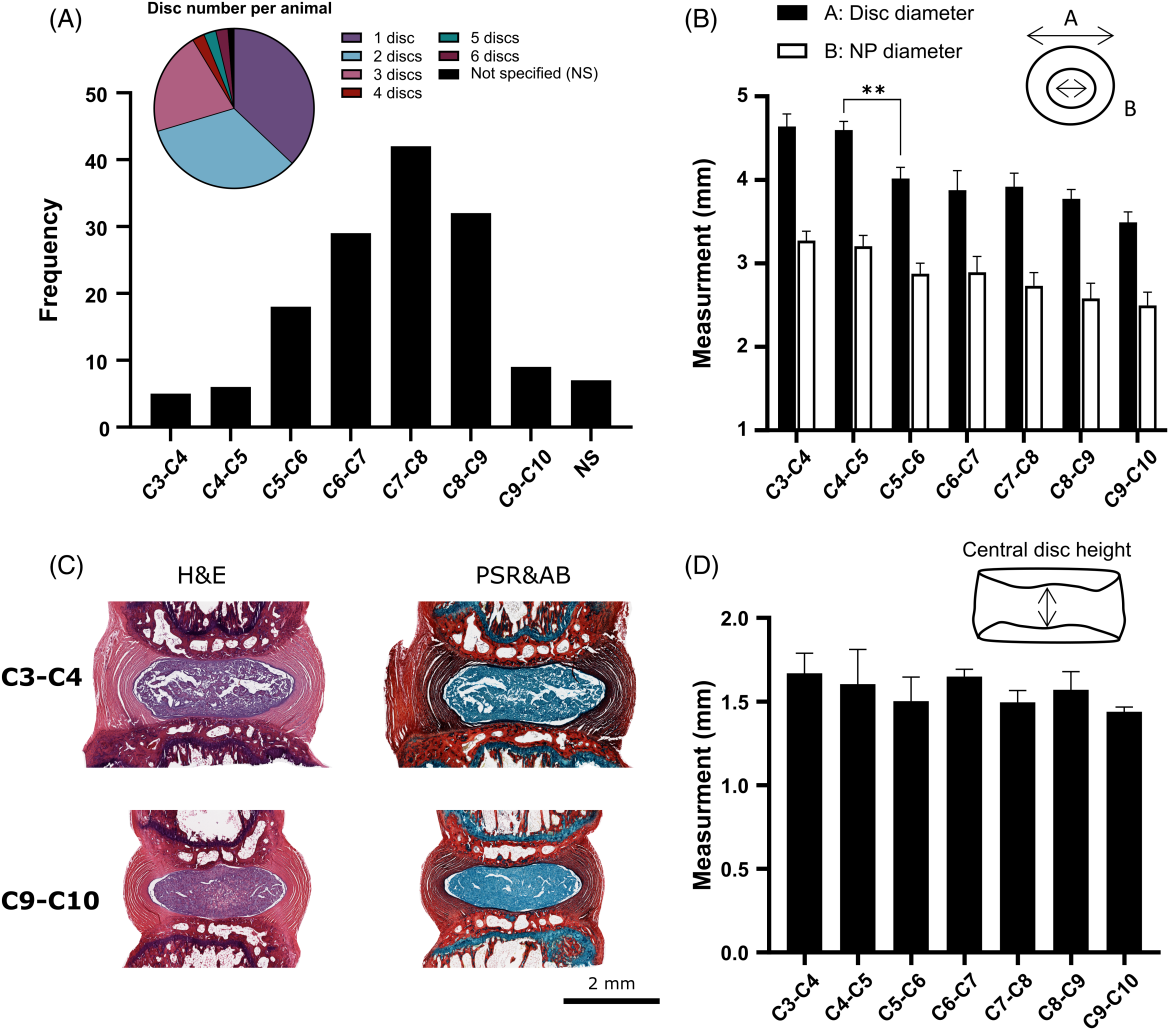
Anatomical and geometrical considerations. (A) The pie chart highlights the number of discs used per animal in each study, with most studies using 1 (37.5%), 2 (33.8%), or 3 (21.3%) discs. The bar graph shows the frequency and range of caudal levels used across these studies, with C7–C8 being the most frequent level and seven studies not specifying (NS) which discs were used. (B) Experimentally measured external disc diameter and internal nucleus pulposus (NP) diameter for 8‐week‐old Wister rats (*N* = 6). Statistics are only shown in the case of a statistical difference between adjacent levels with *p* = 0.0048. (C) Sagittal histology sections showing hematoxylin and eosin (H&E) and picrosirius red (PSR) combined with alcian blue (AB) for the most proximal and distal caudal discs investigated. (D) Experimentally measured central disc height, with no statistical significance found between adjacent levels within this range

Disc geometry and scale is an important parameter to consider in animal models, particularly when relating preclinical results back to the human IVD. Furthermore, it may be important within the rat tail model design itself, for example, when comparing between a treatment and a control disc at different caudal levels. Figure 3A highlights the number of discs used per animal and the frequency of each caudal level across the reviewed studies. Over 90% of the papers used three discs or fewer in their study, with 30 of 80 (37.5%) using one disc, 27 of 80 (33.8%) using two discs, and 17 of 80 (21.3%) using three discs per tail. Less than 8% used more than three discs, with six discs per animal being the greatest number reported. The caudal level reported in the literature ranged from C3–C4 to C9–C10. Despite seven studies not reporting the exact caudal levels used, there is a clear inclination toward discs between C5–C6 and C8–C9, with C7–C8 being the most frequently used disc (42 studies). We have carried out a geometrical analysis of rat tails (8‐week‐old Wistar rats [*N* = 6]) through histological slices in the transverse and sagittal plane. Figure 3B shows the full disc diameter and the NP diameter determined from the transverse plane. Moving distally along the rat tail there is a trend of decreasing disc size, with the diameter of C3–C4 more than 1 mm larger than the diameter of C9–C10. Although this may appear quite a small difference, when relating diameter to volume, this results in a 40%–60% decrease in disc volume (depending on disc height). A statistically significant difference between adjacent caudal levels was only found for the disc diameter between C4–C5 and C5–C6 (*p* = 0.0048). This is particularly interesting as it correlates well with the fact that most studies use discs within the C5–C6 to C8–C9 range, where our data suggests no significant difference in geometry among these discs. Figure 3C shows a representative image of hematoxylin/eosin and picrosirius red/alcian blue staining of the most proximal and distal discs included in this review. Figure 3D suggests very small differences in disc height between C3–C4 and C9–C10, with no statistical significance observed. However, from our experience larger decreases in disc height become rapidly apparent beyond disc C9–C10, with C10–C11 showing a significant difference in disc height compared to more proximal levels (data not shown as these discs were out of the caudal range reported in the literature and thus not pertinent to this review). Comparing this geometrical analysis to caudal discs from other species, we previously found little difference in bovine caudal disc diameter in the most proximal discs (C1–C2 to C5–C6), with differences only becoming significant at C6–C7. This is slightly more distal in the bovine tail than what was detected in the rat. However, unlike the rat analysis, significant differences in the central disc height of bovine caudal discs were found throughout the range C1–C2 through to C7–C8.^27^

## 4 INDUCTION OF DEGENERATION

### 4.1 Commonly reported anesthetics and considerations

**FIGURE 4:**
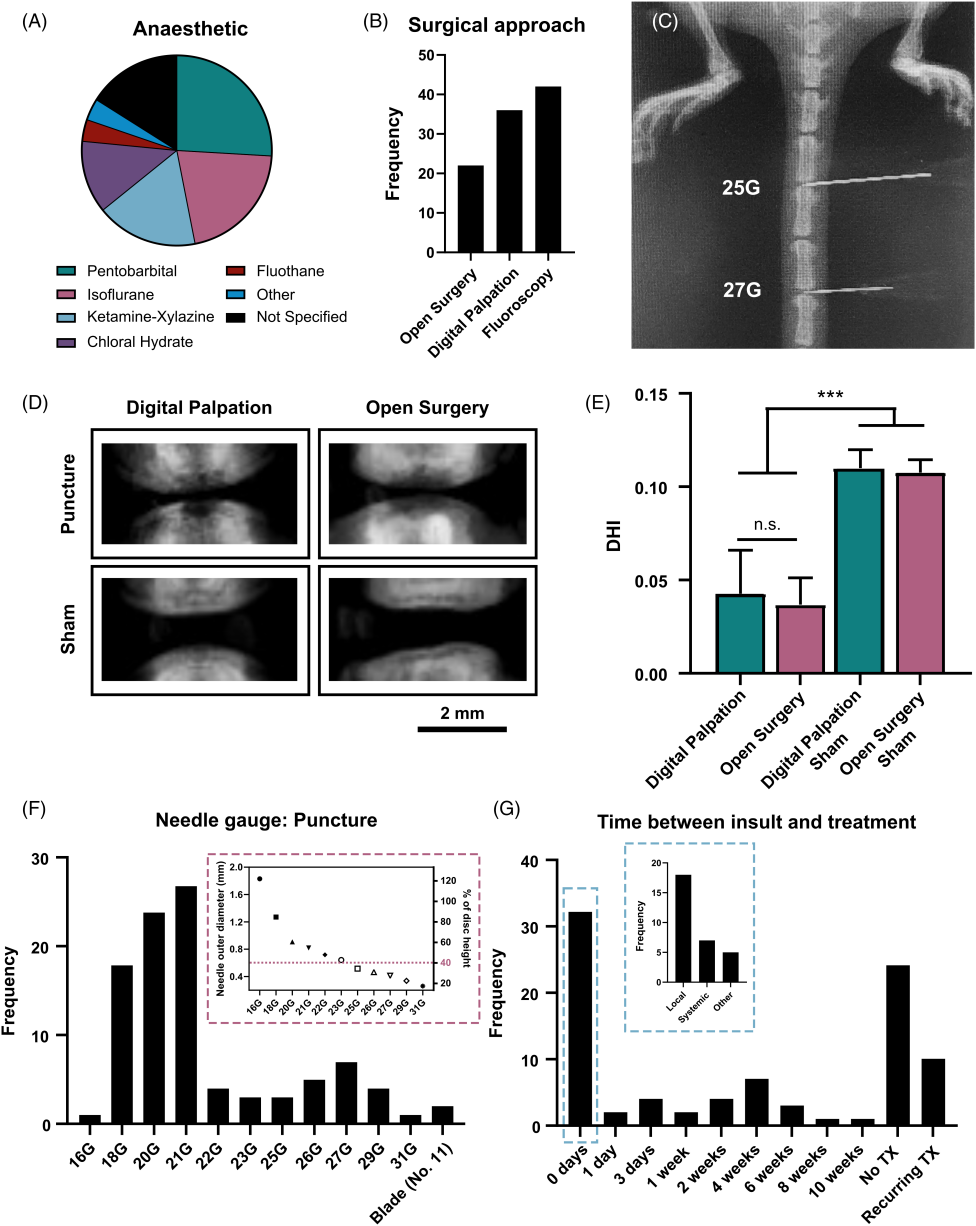
Surgical approaches to establishing a degeneration rat tail model. (A) The majority of studies used pentobarbital (21/80 = 26.3%), isoflurane (17/80 = 21.3%), or ketamine–xylazine (14/80 = 17.5%). Less than 20% of studies used an alternative anesthetic; chloral hydrate, fluothane or other (medetomidine, midazolam, butorphanol, pelltobarbitalum natricum, or atipamezole). However, 13 studies (16.1%) did not report the anesthetic used. (B) The frequency of either digital palpation, open surgery, or fluoroscopically guided punctures used across the reviewed studies. (C) A microcomputed tomography (μCT) image demonstrating the fluoroscopically guided approach used to confirm the needle position. (D) μCT images to observe the reduction in disc height 2 weeks following subcutaneously puncture through digital palpation and open surgery. (E) Quantitative analysis using disc height index (DHI) to compare the effect of percutaneous puncture and open surgery (*p* < 0.003). (F) The frequency of needle gauges used to puncture the disc across the reviewed studies. The inset graph relates the outer needle diameter of each needle gauge to disc height, assuming an average disc height of 1.5 mm (G) The time between puncture insult and treatment (TX) across the reviewed studies

Minimizing anesthetic‐related side effects is important both for animal welfare as well as reducing potential experimental interference or confounding factors.^28^ Whether an inhalable or injectable anesthetic is used may be dictated by the experimental setup. In the case that injection is the preferred method, it is often recommended that either subcutaneous or intraperitoneal routes be used, as intramuscular injections may result in tissue irritation and may adversely affect animal well‐being. As shown in Figure 4A, the three most reported anesthetic agents were pentobarbital (21/80 = 26.3%), isoflurane (17/80 = 21.3%), and ketamine–xylazine (14/80 = 17.5%). However, studies have demonstrated that ketamine–xylazine could induce abnormally slow heart rates and low arterial blood pressure when compared to pentobarbital, and lower heart rate and respiratory rate when compared to isoflurane.^28^, ^29^ It has further been suggested that isoflurane had a nearly fivefold faster anesthetic induction time, a 2.5‐fold faster recovery time, and a deeper anesthetic state than ketamine–xylazine as evidenced by fewer incidences of toe pinch responses while anesthetised.^29^ An additional advantage of inhalable anesthetics over injectables is the ability to rapidly modify anesthetic exposure during the procedure.

### 4.2 Puncture induced degeneration model

As shown in Figure 4B, three surgical approaches were reported: open surgery (22/80 = 27.5%), digital palpation of the IVD (36/80 = 45.0%), or fluoroscopically guided punctures (42/80 = 52.5%). However, it is important to note that studies often use a combination of these approaches. Fifteen studies used digital palpation and then confirmed the puncture using fluoroscopy while three studies carried out an open surgery under fluoroscopy. For illustrative purposes, Figure 4C, demonstrates the fluoroscopically guided approach with microcomputed tomography (μCT) used to confirm the puncture of two caudal discs with a 25G and 27G needle. When using fluoroscopy, disc levels can be identified by imaging and counting down from the sacrum (with C1 being the first “free” vertebra following the four sacral vertebrae). If digital palpation is the preferred approach, disc levels can be identified by locating the processes along the vertebrae, with the last set of palpable processes reportedly found on the fifth caudal vertebra (i.e., level C5).^30^ When conducting a pilot study to investigate puncture and treatment approaches, we compared open surgery with the disc exposed to percutaneous needle puncture through digital palpation to evaluate whether these two methods exhibited any differences. All procedures for this study were approved by the animal research ethics committee of Trinity College Dublin and the Health Products Regulatory Authority in Ireland (Approval‐AE19136/P149). Briefly, 12‐week‐old Wistar rats (*n* = 4; 2 male and 2 female) were anesthetized with 2%–4% isoflurane with 1%–2% O_2_. Meloxicam (1.5 mg/kg) was administered subcutaneously 1–2 h preoperatively. For the open surgery group, three caudal discs (C4–C5 through to C6–C7), in one male and one female rat, were exposed with a ~3 cm incision on the posterior caudal plane using a size 21 blade. Discs C4–C5 and C6–C7 were then punctured using a 21G needle, leaving disc C5–C6 intact. The puncture process involved inserting the needle intradiscally, rotating it 180 degrees, and holding it for 5 s before removing the needle. The skin was then sutured using 4‐0 nylon sutures, animals were given a subcutaneous injection of buprenorphine (0.05 mg/kg), returned to their housing and allowed to recover. For the percutaneous punctures, discs C4–C5 through to C6–C7, for one male and one female rat, were identified via digital palpation and punctured through the skin using a 21G needle in the same manner as the open surgery, but following a percutaneous approach (i.e., without making an incision in the tail). Rats were likewise given a subcutaneous injection of buprenorphine prior to recovery. All rats were given daily injections of meloxicam (1.5 mg/kg) for 3 days following surgery.

Discs were imaged using μCT (Scanco VivaCT 80, 45 kVp, 177 μA, 8 W, and 78 μm voxel size) after 2 weeks to observe disc height (Figure 4D). As shown in Figure 4E, our data suggests that percutaneous punctures led to the same degree of IVD collapse and degeneration over the 2‐week period. However, there was observably less inflammation near the puncture site in the percutaneous group than in the group with the open incision, likely leading to a lower degree of discomfort for the animals. Alternatively, we found that when delivering a treatment into the disc, open surgery allowed for clear visualization of the disc and provided unobstructed confirmation of efficient intradiscal delivery, as well as retention of the therapy within the disc. Animal welfare should always be considered in the design of animal models. Thus, if two procedures are required (e.g., a needle puncture procedure for the induction of degeneration followed later by a second procedure to deliver a local treatment intradiscally), percutaneous puncture guided by digital palpation was found to be sufficient for the induction of degeneration. Then for the follow‐up procedure, open surgery can be used to ensure proper treatment delivery, as mentioned previously. This approach eliminates the need for two open incisions in close temporal proximity of one another. This could be considered a valid refinement in animal welfare by diminishing pain and discomfort for the animal and reducing the potential for surgical complications such as infection.

### 4.3 Selection of needle gauge size

For the induction of degeneration, previous published work suggests that needle gauges smaller than 23G did not produce consistent and significant degrees of degeneration in rat which was confirmed through magnetic resonance imaging (MRI).^31^ Work conducted in a mouse model previously suggested that needle diameters >40% of disc height were necessary to alter disc function in the lumbar spine.^32^ This hypothesis was further investigated in rat models, where similar trends in the loss of mechanical function, loss of signal intensity and MRI indices, and loss in histological structures were observed following punctures with needle gauges ranging from 18G to 27G.^33^, ^34^, ^35^, ^36^ A study by Keorochana et al. further suggested that needle size affects not only the degree of degeneration but also the rate of degeneration.^36^ The authors investigated the effects of 18G, 20G, and 21G needles each in C6–C7 and C7–C8 discs (all >40% of the disc height). The 18G needle puncture promoted significant differences in disc height index at all time points, and greater differences became apparent over time. MRI revealed significant degrees of degeneration at early time points (i.e., 2 weeks) only in the 18G needle puncture group. 20G needles promoted degeneration that became apparent at intermediate time points (4, 6, and 8 weeks), while 22G needle puncture only demonstrated effects at the 8‐week time point. Histological evaluations corroborated those findings.

As highlighted in Figure 4F, we found that a significant majority of studies in our review adhered to these findings, with 28 studies inducing degeneration using a 21G needle, 20 studies with a 20G needle and 17 studies using an 18G needle. One point for discussion is the degree of degeneration desired. Whether mild degeneration or complete destruction of the disc is desired is entirely dependent on the experimental design and the scientific question or objective of the study. The inset graph highlights the outer diameter of each needle gauge, as well as relating it to disc height (assuming a disc height of 1.5 mm) to better inform design choices. For example, assuming an average caudal disc height of 1.5 mm, puncture with a large needle gauge such as an 18G (average outer diameter of ~1.25 mm, ~80% disc height) may lead to complete destruction of the IVD rather than mild degeneration and may thus interfere with studies seeking to provide long‐term disc restabilization or regenerative tissue engineering models. Alternatively, if severe defect formation is needed for the proposed model, puncture with a 27G needle (average outer needle diameter of ~0.4 mm, ~25% disc height) would not likely be sufficient and a larger needle may be necessary.

## 5 TREATMENT OF DEGENERATION

### 5.1 Timepoint and type of treatment

Our review found that 32 studies provided treatment immediately following puncture (Figure 4G). Of those, 18 studies provided a local IVD treatment, while 7 studies focused on systemic delivery such as incorporation of drugs into food, or intraperitoneal injections. Twenty‐four studies provided no treatment but focused on the study of different parameters associated with progression of IVD degeneration. It is worth discussing that whether treatment is delivered following the onset of acute disc degeneration or immediately following puncture may be dependent on the research question being investigating. For example, research on how a therapeutic slows down degeneration and treatments for restoring mechanical function in a degenerative disc are likely to require different experimental setups, and thus the outcomes may be impacted by improper design. Thus, careful consideration of treatment delivery as a factor of research outcomes of interest is of importance.

### 5.2 Needle gauges and volumes delivered

**FIGURE 5:**
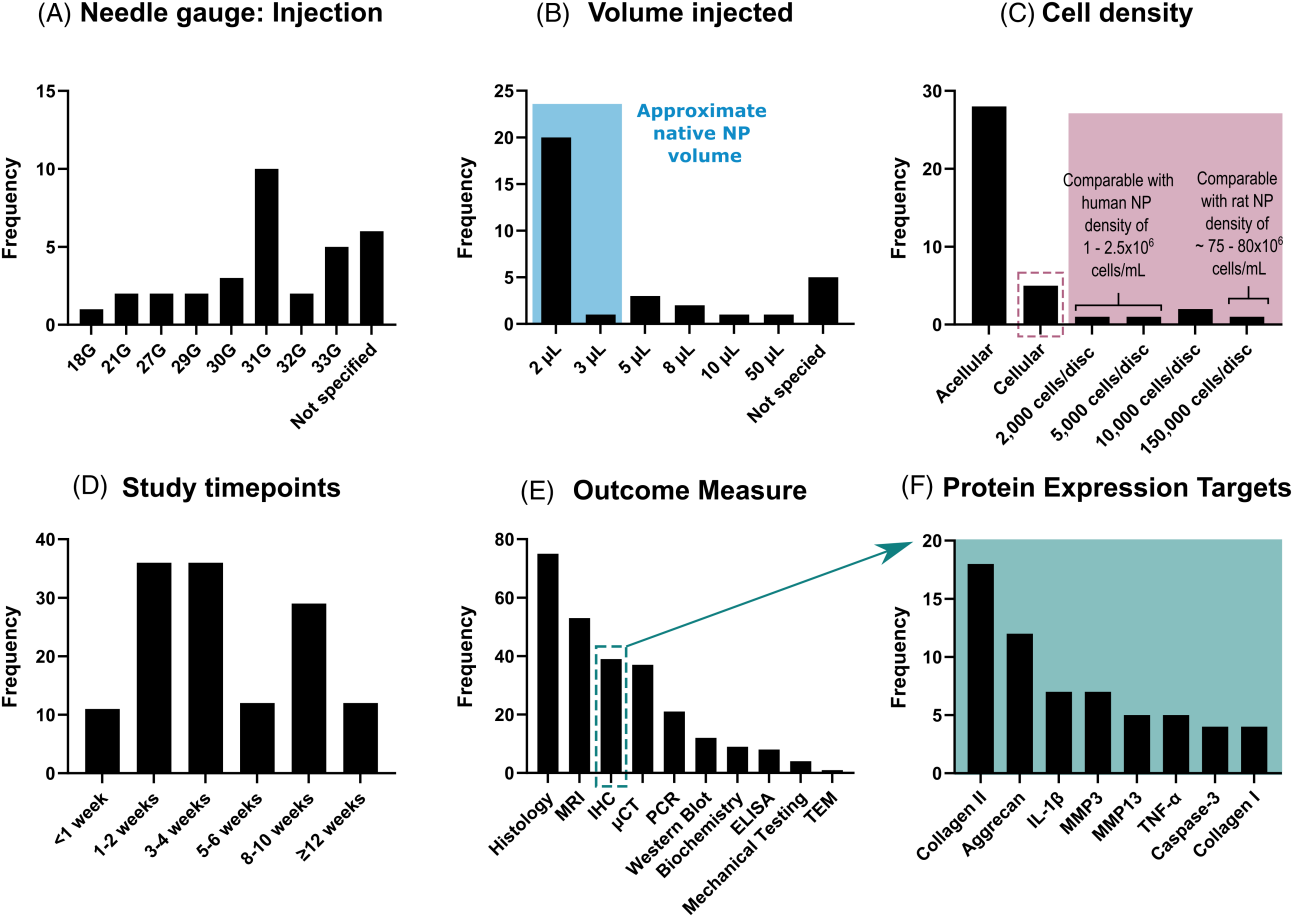
Treatment parameters and outcome measures used in the reviewed rat tail models. (A) Treatments are commonly administered through a 31G needle (10 papers). However, six papers failed to report on the needle gauge used. (B) Most studies reported an injection volume of 2 μl (20 studies), which is within the approximated rat NP volume estimated from NP tissue weights and accounting for hydration, blue overlay. (C) The majority of reviewed studies used an acellular approach (28 studies). Within the studies that used a cell‐based treatment (five studies) the cell densities varied extensively. Assuming a 2 μl NP volume, these cell densities have been compared to the native cell densities of the human and rat NP. (D) The literature reported a range of timepoints, with 1–2 weeks and 3–4 weeks the most popular for early timepoints and >8 weeks typical for later timepoints. (E) The frequency of different outcome measures used throughout the reviewed literature. (F) The most common protein expression targets determined through immunohistochemistry

Treatments are commonly administered through a 31G needle (Figure 5A) as the outer diameter of the needle is <20% of a rat disc height and is unlikely to promote further degeneration.^31^ However, a caveat to this needle diameter is that it may present complications in the delivery of biomaterials, as parameters such as viscosity/density and shear may impact the feasibility of material delivery and extrusion through this needle gauge. This may result in lower gauge (wider bore) needles being better candidates for biomaterial delivery. Focusing on the studies that provided a local treatment delivery into the intradiscal space, Figure 5B shows that most studies reported an injection volume of 2 μl (20 studies). To better estimate NP volume, we obtained six caudal spines from freshly euthanised Wistar rats (male, 20 weeks old) and extracted the NP of six discs per rat (C4–C5 through to C9–C10) by cutting the discs along the transverse plane at the superior CEP, and then removed the gelatinous NP tissue using needle‐point forceps. We weighed the extracted NP tissue and measured 3.1 ± 0.5 mg per disc. Assuming hydration ranges between 70% and 90%, this would roughly equate to volumes between 2 and 3 μl. It may thus follow that the reported injected volumes of 2–5 μl may be best suited for intradiscal treatment delivery without causing detrimental changes in intradiscal pressure.

### 5.3 Cellular versus acellular treatment

Again, focusing on the studies that provided a local treatment delivery into the intradiscal space, 28 studies used an acellular approach and 5 used a cell‐based treatment. Although two studies compared both a cellular and an acellular approach. Figure 5C highlights that among the cellular approaches, there does not appear to be a consensus on the cell number to be delivered as within our sample size of five reviewed papers, we observed values ranging from 2000 to 150 000 cells/disc (assuming a NP or injection volume of 2 μl; this range is equivalent to 1 million to 75 million cells/ml). It is typically reported that the native human NP cell densities is ~4 million cells/ml.^37^ However, more recent work assessing cell density with respect to degeneration grade have reported the total NP cell density to be closer to ~2.5 million cells/ml (depending on specific grade of interest) and out of the total cell population only ~1.3 million cells/ml appear to be metabolically active.^38^ In comparison, native NP cell densities for rat discs have been reported as high as 80 million cells/ml in adult Sprague Dawley rats.^39^ This suggests that more deliberate consideration is needed in the design of cellular treatments. It is important to assess whether the aim is to fully replace the NP with a biomaterial which matches the native density of the tissue, or whether the therapeutic aims to aid and stimulate inherent regeneration of the native NP. In the case of the latter, it may be important to consider the total cell number already resident in the rat NP and how many more cells can be implanted without further exacerbating the degenerative niche and in turn affecting the viability and regenerative potential of the treatment.^40^ Furthermore, given the significant differences in rat and human NP cell density, it may be important to take this into consideration at an earlier stage of design and testing, to assist not only in the progression to large animal models but to help realize more successful clinical translation.

## 6 PRIMARY OUTCOME MEASURES

According to Figure 5D a similar number of studies evaluated the outcome measures at 1–2 weeks (36 studies), 3–4 weeks (36 studies), and >8 weeks (41 studies). It is worth noting that most studies used multiple timepoints to allow for temporal comparisons. These timepoints are likely recurrent as they may be representative of the progression of pathology from acute to intermediate and chronic stages of IVD degeneration.^41^

Figure 5E shows the frequency of different outcome measures used throughout the reviewed literature. For longitudinal or temporal studies, where in vivo data was to be collected throughout the study, MRI (53 studies) and μCT (37 studies) are the predominant outcome measures reported in the literature. Although these measures were also popular in terminal timepoint studies. However, in postmortem, histological analysis (75 studies) was the most popular evaluation metric. Immunohistochemistry (39 studies) was another popular approach, as it provides a level of biologically relevant detail that is not necessarily attainable with standard histological techniques. Furthermore, recent work by Lai et al. proposed a standardized method for histopathology scoring, which may further support the path toward consistent experimental development.^42^ Within the subset of papers that employed immunohistochemistry as an outcome measure, Figure 5F, the most popularly reported targets were associated with either extracellular matrix deposition, specifically collagen II (18 studies), aggrecan (12 studies), MMP3 (7 studies), and MMP13 (5 studies), or markers associated with an inflammatory response, specifically IL‐1β (7 studies) and TNF‐α (5 studies). While tissue biomechanics are an important factor to consider in tissue regeneration approaches, only four reviewed works reported mechanical testing data. This is likely due to these experiments most often being utilized in organ culture models due to reduced confounding factors and a subsequent ability to obtain clearer outputs. Radiographic, MRI, and histological analyses all captured the progression of degeneration as evidenced by differences in disc height and structure compared to control discs, as early as 2 weeks.

## 7 ASSOCIATED LIMITATIONS WITH RAT TAIL MODELS

The objective of this review is to provide a summary of the parameters most employed to establish a rat caudal degeneration model. One significant advantage of the caudal model is the ease of access and high throughput nature. It can help in the interpretation of data as results are less likely to be confounded by the loadings observed in the lumbar spine, particularly at an early stage of therapeutic development, before transitioning to a larger, more anatomically relevant animal model. However, limitations do exist. For example, lumbar models offer more biological complexity, and more physiological relevance in terms of biomechanical parameters. Thus, lumbar models may be more useful to researchers seeking to study neoinnervation and vascularization or subsequent immune or inflammatory responses and their associations with the pain phenotype. Caudal models may present challenges as exposure to neural and vascular microenvironments differs from other anatomical regions such as the lumbar spine. As mentioned previously disc scale is an important parameter to consider in rat models, particularly when relating preclinical results back to the larger avascular human IVD with its unique microenvironment,^40^ which is often speculated to be linked to the high failure of perspective studies.^43^, ^44^, ^45^ Correlation of metrics from rat models toward human applications is difficult due to a number of characteristic differences between the two. For example, a key difference can be observed in terms of cell populations. Humans have been observed to undergo a significant transition of notochordal cells toward nucleopulpocytes, resulting in a near depletion of notochordal cells by age 10.^2^, ^14^, ^46^ However, these changes are less abrupt in rat IVDs, with populations of notochordal cells still being observed into skeletal maturity and adulthood.^14^, ^47^ Another key difference involves vascularization at early stages of development. In humans, significant decreases in vascularization have been observed in childhood as early as age 4, with the discs becoming further avascular with aging.^39^, ^48^ By contrast, while a decrease in vascular body presence was likewise observed with aging in rats, this was not observed until late adulthood (22 months in rats).^49^ Taken together, these factors suggest that more in depth knowledge is needed on the microenvironmental niche created within these rat tail models, particularly in the case of nutrient demands and availability to ensure that cell‐based therapies are being tested under robust and clinically relevant conditions. Finally, another limitation involves the study of the pain phenotype. While some studies (9/80 = ~11%) discussed a focus on the quantification of pain‐related markers such as differential cytokine levels, only one study among all reviewed papers conducted behavioral analysis for pain‐related metrics. Behavioral analysis is often reported in lumbar models of radiculopathy but are less prominent in caudal models due to the reduced nerve presence by comparison. Nevertheless, due to the importance of gaining a better understanding of the pain phenotype, this may be a significant limitation of using caudal models.

## 8 CONCLUSION

Rat caudal models of disc degeneration have demonstrated significant utility in the study of disease progression and its impact on tissue structure, composition, and mechanical performance. While different models may yield different experimental outcomes, a considerable number of studies utilize a physical insult by means of needle puncture to induce degeneration. Although previous work, such as that by Elliott et al.,^32^ have provided meaningful information toward consistent and reproducible induction of degeneration in rodent models, the literature still maintains significant variability in these approaches. Caudal models offer benefits in terms of surgical procedures since a less invasive approach is needed for a caudal disc puncture compared to other anatomical regions. The use of caudal models further enables the researcher to create percutaneous tissue insults to initiate disc degeneration without the need for an open surgery, which in turn, may improve overall animal well‐being and decrease the risk for surgical complications.

**FIGURE 6:**
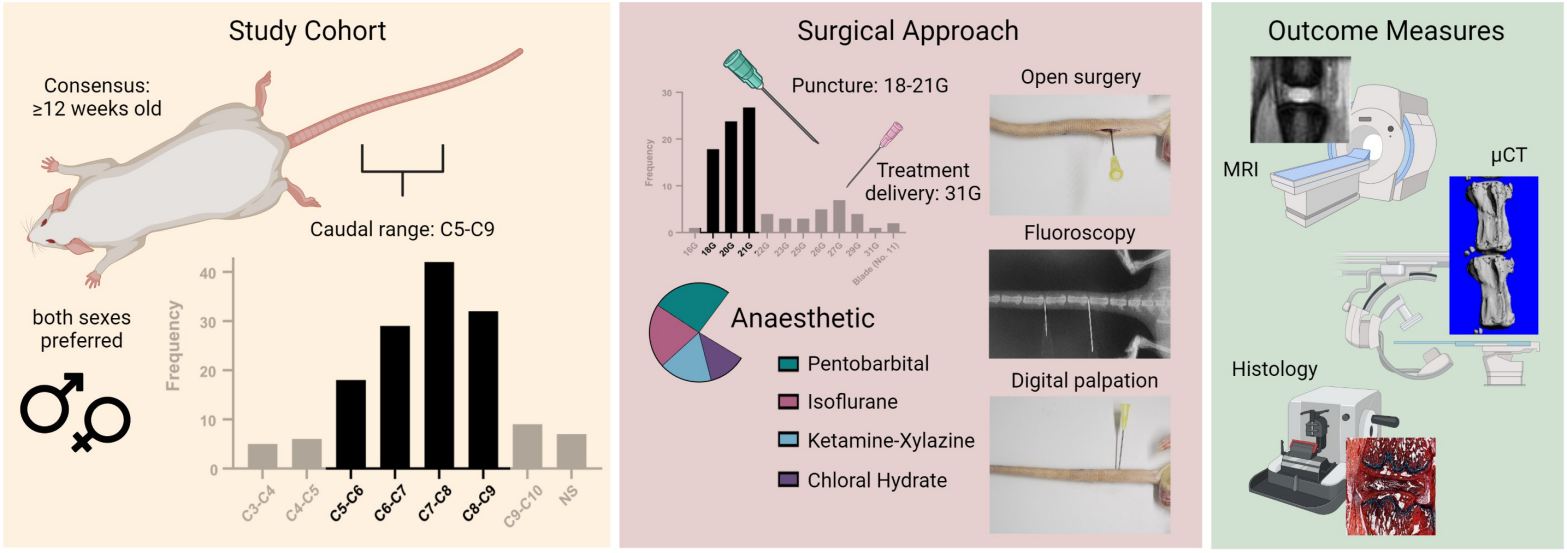
A visual summary of the main findings compiled from the reviewed rat tail models. There is a general consensus to use rats at least 12 weeks old to ensure skeletal maturity, and both sexes are preferred. Most studies operate within the C5–C9 range. 18–21G needles are preferred for inducing degeneration, while a 31G needle is preferred for intradiscal treatment delivery. Pentobarbital and isoflurane are the favored anesthetics, followed by ketamine–xylazine and chloral hydrate. The surgical approach may be based on the specific experimental design and can include open surgery, fluoroscopy, and digital palpation. The most reported outcome measures were magnetic resonance imaging (MRI), microcomputed tomography (μCT) and histology. Created with BioRender.com

Figure 6 visually summarizes the main findings of this review. In the case that multiple surgical procedures are required, as is the case for experiments where the induction of degeneration and its treatment are temporally distinct, induction of degeneration using a percutaneous puncture is recommended as it may lead to improved animal welfare overall. For the intradiscal delivery of a treatment group, an open surgery may be the best approach as it allows for full visualization and confirmation of effective delivery into the disc. The use of a 31G needle for treatment delivery was the most popular choice, although needle gauges as large as 27G have been used without observable or significant changes to the disc structure or contribution to disease progression. These ranges may be beneficial for drug‐based treatments, but more consideration may be necessary when delivering viscous biomaterials as their extrusion may become hindered at higher gauges.

For the induction of degeneration via needle puncture, different approaches may be selected. Needle gauges of 25G–23G (~0.5–0.6 mm outer diameter and ~35%–45% of the disc height, respectively) may be best suited for the induction of acute degeneration in accordance with size ratios previously reported in the literature, while gauges of 21G–18G (~0.8–1.3 mm outer diameter and ~55%–85% of the disc height, respectively) may be better suited for a more aggressive defect. Likewise, time between insult and treatment may depend on the specific research question as the degree of degeneration within the disc prior to delivery of a treatment will have significant impact in the experimental outcomes. Furthermore, delivery of intradiscal volumes between 2 and 5 μl is advised, as this range falls within the observed and reported dimensions of NP tissue (with a margin for error) and would allow for effective delivery with minimal effects on intradiscal pressure. For the length of treatment application following induction of degeneration, timepoints of 1–2, 3–4, and 8–10 weeks were most often selected as measures of early, intermediate, and late‐stage disc degeneration.

## AUTHOR CONTRIBUTIONS

All authors contributed to the conception and design of the work. Marcos N. Barcellona, Emily E. McDonnell, and Shani Samuel performed the acquisition, analysis, and interpretation of literature data. Marcos N. Barcellona and Emily E. McDonnell preformed the pilot rat study, acquisition, analysis, presentation, and interpretation of results, drafting of the article, revising it critically and final approval. Conor T. Buckley, as the overall project funding holder, takes responsibility for the integrity of the work from inception to finalized article, provided substantial contribution to data interpretation and presentation, drafting of the article, revising it critically, and final approval.

## CONFLICT OF INTEREST

The authors declare no conflicts of interest.

## Supporting information


**Table S1** List of reviewed manuscripts and relevant experimental details extracted from the literature
