# Supplementary material for: Rat tail models for the assessment of injectable nucleus pulposus regeneration strategies
Source: JOR Spine. 2022 Jul 11;5(3):e1216. doi: 10.1002/jsp2.1216 (PMC9520766; doi:10.1002/jsp2.1216)
Supplement: Supplementary file 1 — Table S1 List of reviewed manuscripts and relevant experimental details extracted from the literature [file JSP2-5-e1216-s001.pdf]

DOI	Author and Year	Title	Number of rats	Age (weeks); Sex (M/F)	Strain	Anesthetic	Number of discs punctured; disc level	Procedure approach	Time from induction of degeneration to treatment	Treatment details (if applicable)	Time from degeneration to euthanasia	Outcome measures
10.1016/j.jot.2021.04.003	Wu et al., 2021	Krüppel like factor 10 prevents intervertebral disc degeneration via TGF-β signaling pathway both in vitro and in vivo	24	12; M	Sprague-Dawley (SD)	Chloral hydrate	1; C7-C8	Digital palpation, percutaneous puncture with 21G needle to depth of 5 mm	Immediately after insult	Intradiscal; 10 μL, no needle gauge specified	Euthanasia 8 weeks after initial puncture	X-ray, MRI, histology, immunohistochemistry (IHC)
10.1002/ar.24519	Fattah & El-Din, 2021	Granulocyte-colony stimulating factor improves intervertebral disc degeneration in experimental adult male rats: A microscopic and radiological study	36	12; M	SD	Ketamine-Xylazine	2; C5-C6, C7-C8	Open surgery, puncture with 20G needle to depth of 5 mm	6 weeks after insult	Systemic (subcutaneous treatment delivery)	Euthanized 8 weeks after puncture	X-ray, histology, IHC, TEM
10.1155/2021/6672978	Dai et al., 2021	Salvianolic Acid B Protects Intervertebral Discs from Oxidative Stress-Induced Degeneration via Activation of the JAK2/STAT3 Signaling Pathway	60	No specified age or weight; M	SD	Fluothane in oxygen/nitrous oxide	1; C8-C9	Digital palpation, percutaneous disc puncture with 20G needle to depth of 5 mm	Treatment delivered following surgery	Systemic (oral gavage)	Euthanized 6 weeks after puncture	MRI, histology, biochemical analysis, ELISA, western blotting
10.1155/2021/6631562	Zhang et al., 2021	Quercetin Alleviates Intervertebral Disc Degeneration by Modulating p38 MAPK-Mediated Autophagy	36	8; M	SD	Pentobarbital	no details	Fluoroscopy guided percutaneous puncture with 27G needle to depth of 4 mm	Immediately after insult	Systemic (intragastric delivery)	Euthanized 8 weeks after puncture	MRI, histology, IHC, western blotting
10.1155/2021/5510124	Tian et al., 2021	Intervertebral Disc Degeneration Induced by Needle Puncture and Ovariectomy: A Rat Coccygeal Model	Stage 1: 36; Stage 2: 9	12; F	SD	Pentobarbital	3; C5-C6 to C7-C8	Open surgery, puncture with 21G needle to depth of 3 mm	Immediately after insult	Systemic (subcutaneous delivery)	Euthanized at 4, 8 and 12 weeks after puncture	Micro-MRI, histology
10.1155/2021/5556122	Dai et al., 2021	Sodium Tanshinone IIA Sulfonate Ameliorates Injury-Induced Oxidative Stress and Intervertebral Disc Degeneration in Rats by Inhibiting p38 MAPK Signaling Pathway	40	12; M	SD	Fluothane in oxygen/nitrous oxide	1; C8-C9	Digital palpation, percutaneous disc puncture with 20G needle to depth of 5 mm	Immediately after insult	Systemic (intraperitoneal delivery)	Euthanized 4 weeks after puncture	X-ray, MRI, histology, IHC, ELISA, western blotting
10.1002/jor.24757	Nakashima et al., 2020	Quantitative analysis of intervertebral disc degeneration using Q-space imaging in a rat model	15	8; F	Wistar (W)	N/A	4; C4-C5 to C7-C8	Open surgery, puncture with 23G needle	Started 1 week prior to insult	Systemic (oral administration)	Euthanized 5 months after puncture	MRI, histology
10.1016/j.spinee.2020.04.024	Deshmukh et al., 2020	A small-molecule inhibitor of the Wnt pathway, lorecivivint (SM04690), as a potential disease-modifying agent for the treatment of degenerative disc disease	18	10; no sex specified	SD	Ketamine-Xylazine	2; C8-C9, C9-C10	Digital palpation + fluoroscopy, percutaneous puncture with 20G needle	1 week	Intradiscal; 2 μL, no needle gauge specified	Euthanized 8 weeks after puncture	X-ray, histology
10.1002/jbm.b.34541	Hu et al., 2020	Thermosensitive hydrogels loaded with human-induced pluripotent stem cells overexpressing growth differentiation factor-5 ameliorate intervertebral disc degeneration in rats	24	300 g (~8-10 week); M	No details	Ketamine	3; C6-C7 to C8-C9	Fluoroscopy guided percutaneous puncture with 21G needle to depth of 5 mm w/ negative pressure (suction)	At the time of insult	Intradiscal; 2 μL, no needle gauge specified, cell delivery at 1x10^4/disc	Euthanized at 1, 2 and 3 months after transplantation	X-ray, MRI, histology
10.1152/ajpcell.00271.2019	He et al., 2020	P14ARF inhibits regional inflammation and vascularization in intervertebral disc degeneration by upregulating TIMP3	85	12; M	SD	Pentobarbital	1; C6-C7	Open surgery, puncture with 20G needle	1 week	Intradiscal, 2 μL through 33G needle	Euthanized 9 weeks after induction of degeneration	Histology, RT-qPCR, western blotting, IHC
10.2147/DDDT.S274812	Wang et al., 2020	Acacetin Alleviates Inflammation and Matrix degradation in Nucleus Pulposus Cells and Ameliorates Intervertebral Disc Degeneration in vivo	18	8; M	SD	Pentobarbital	2; level not specified	Digital palpation, percutaneous puncture with 21G to depth of 5 mm	Treatments delivered weekly	Systemic (IP delivery)	Euthanasia at 4 weeks after initial puncture	MRI, histology
10.1155/2020/8319516	Bi et al., 2020	Antiaging factor klotho retards the progress of intervertebral disc degeneration through the toll-like receptor 4-NF-kB pathway	12	12; M	SD	Pentobarbital	1; no details	Digital palpation + fluoroscopy, percutaneous puncture with 27G needle	siRNA delivered 1 day before IDD operation, and twice a week afterwards	Systemic (intravenous injection)	Euthanasia at 2 weeks after initial puncture	Histology, RT-PCR, western blotting
10.7150/THNO.47723	Zheng et al., 2021	A thermosensitive, reactive oxygen species-responsive MR409-encapsulated hydrogel ameliorates disc degeneration in rats by inhibiting the secretory autophagy pathway	10	12; F	SD	Pentobarbital	1; C7-C8	Fluoroscopy guided percutaneous puncture with 29G needle to depth of 2 mm	No treatment	No treatment	Euthanasia at 8 weeks after initial puncture	MRI, X-ray, histology, IHC

		The above paper had two separate animal models: one for induction of degeneration as outlined above, one for hydrogel delivery	50	12; F	SD	Pentobarbital	1; C7-C8	Fluoroscopy guided percutaneous puncture with 29G needle to depth of 2 mm	Treatment delivered at the time of insult	Intradiscal; 3 μL through 29G needle	Euthanasia at 8 and 12 weeks post puncture	X-ray, MRI, histology, IHC
10.1155/2020/6660429	Qin et al., 2020	Danshen attenuates intervertebral disc degeneration via antioxidation in SD rats	60	12; M + F	SD	Fluothane in oxygen/nitrous oxide	1; C8-C9	Digital palpation, percutaneous puncture with 20G through tail	Treatment delivered immediately following puncture	Systemic (oral administration)	Euthanasia 4 weeks after operation	X-ray, MRI, histology, biochemical analysis, IHC, western blotting
10.12998/wjcc.v8.i16.3431	Su et al. 2020	Application of molybdenum target X-ray photogrpahy in imaging analysis of caudal intervertebral disc degeneration in rats	21	10-12; M	SD	Chloral hydrate	1; C8-C9 or C9-C10	Digital palpation, percutaneous puncture with 18G needle	No treatment	No treatment	2-3 weeks	MRI, conventional X-ray, molybdenum target plain X-ray
10.1002/jsp2.1069	Chan et al. 2019	Pulsed electromagnetic fields reduce acute inflammation in the injured rat-tail intervertebral disc	72	12-16; F	SD	Isoflurane	3; C6-C7 to C8-C9	Fluoroscopy guided percutaneous puncture with 20G needle	Treatment started immediately following surgery	Treatment by exposure to pulsed electromagnetic field	4 or 7 days post insult	Histology, RT-PCR, ELISA
https://pubmed.ncbi.nlm.nih.gov/31337166/	Qian et al., 2019	Selection of the optimal puncture needle for induction of a rat intervertebral disc degeneration model	24	12; M	SD	Chloral hydrate	3; C7-C8 to C9-C10	Fluoroscopy guided percutaneous puncture with 16, 18, or 26G needles	No treatment	No treatment	Sacrificed at 1, 2, or 4 weeks post-insult	MRI, histology, IHC
10.1007/s00586-019-05924-3	Li et al., 2019	Diffusion kurtosis imaging provides quantitative assessment of the microstructure changes of disc degeneration: an in vivo experimental study	21	12; F	SD	N/A	1; C6-C7 or C7-C8	Fluoroscopy guided percutaneous puncture with 21G needle to depth of 5 mm	No treatment	No treatment	Up to 14 days	MRI (diffusion weighted and diffussion kurtosis imaging), histology
10.1016/j.jos.2018.08.006	Zhang et al., 2019	Effect of hyperlipidaemia to accelerate intervertebral disc degeneration in the injured rat caudal disc model	30	8-10; M	W	N/A	1; C7-C8	Open surgery, puncture with 20G needle	Diet modified 8-weeks prior to surgery	Systemic (dietary changes)	8 weeks from puncture	Histology, IHC, western blotting, RT-qPCR
10.1155/2019/7189854	Liu et al., 2019	Aspirin-mediated attenuation of intervertebral disc degeneration by ameliorating reactive oxygen species in vivo and in vitro	40	12; M	SD	Pentobarbital	2; C8-C9, C9-C10	Digital palpation + fluoroscopy guided percutaneous puncture with 20G needle to depth of 5 mm	3 days post puncture	Intradiscal; 2 μL through 33G	7 days following insult	X-ray, MRI, histology, IHC
10.1186/s13075-019-1986-8	Zhan et al., 2019	Long non-coding RNA HOTAIR modulates intervertebral disc degenerative changes via Wnt/B-catenin pathway	10	12; M	SD	Ketamine/ketamine hydrochloride	3; C6-C7 to C8-C9	Digital palpation + fluoroscopy guided percutaneous puncture with 21G needle	At the time of insult	Intradiscal; 2 μL through 31G	Euthanasia 4 weeks after puncture	X-ray, MRI, histology
10.1016/j.intimp.2018.10.024	Fang et al., 2018	Wogonin mitigates intervertebral disc degeneration through the Nrf2/ARE and MAPK signaling pathways	48	150 g (~6 week); M	SD	Pentobarbital	1; C7-C8 or C8-C9	Fluoroscopy guided percutaneous puncture with 20G needle to depth of 5 mm	At the time of insult and repeated weekly	Intradiscal; 2 μL through 31G	8 weeks after initial injection	MRI, histology
10.1038/s41598-018-35011-4	Matta et al., 2018	NTG-101: A Novel Molecular Therapy that Halts the Progression of Degenerative Disc Disease	27	12; F	W	N/A	5; no details	Fluoroscopy guided percutaneous puncture with 27G	4 weeks	Intradiscal; 8 μL through 32G	10 weeks post insult	Histology, IHC
10.1016/j.actbio.2018.07.008	Moriguchi et al., 2018	In vivo annular repair using high-density collagen gel seeded with annulus fibrosus cells	42	10-12; M	Athymic nude (AN)	N/A	1; C3-C4	Open surgery, puncture with 18G needle	At the time of insult	Focus on annular repair model	2 weeks and 5 weeks	MRI, X-ray, histology
10.1016/j.jot.2018.07.008	Ni Li et al., 2018	Disc degeneration promotes regional inhomogeneity in the trabecular morphology of loaded rat tail vertebrae	30	12; M	SD	Pentobarbital	2; C8-C9, C9-C10	Digital palpation percutaneous puncture with 18G needle	No treatment	No treatment	1 week	X-ray, MRI, histology
10.12659/MSM.910636	Chen et al., 2018	Time-course investigation of intervertebral disc degeneration induced by different sizes of needle punctures in rat tail disc	36	16-24; no sex specified	SD	Chloral hydrate and isoflurane	3; C5-C6, C7-C8, C9-C10	Fluoroscopy guided percutaneous puncture with 18, 21, or 25G needle	No treatment	No treatment	2, 4 and 6 weeks	X-ray, MRI, histology, RT-PCR
10.1038/s41419-017-0151-z	Wu X et., 2018	Prolactin inhibits the progression of intervertebral disc degeneration through inactivation of the NF-κB pathway in rats article	80	12; M	SD	Chloral hydrate	2; C7-C8, C8-C9	Digital palpation percutaneous puncture with 20G needle	At the time of insult	Intradiscal; 2 μL through 33G	4,7,14,28 days	X-ray, MRI, histology, IHC

10.1002/jor.23628	Hu et al., 2018	Optimization of puncture injury to rat caudal disc for mimicking early degeneration of intervertebral disc	Stage 1: 12; Stage 2: 30	12; no sex specified	W	Pentobarbital	Stage 1: 6, no details; Stage 2: 3, C6-C7 to C8-C9	Digital palpation + fluoroscopy guided percutaneous puncture with 18, 21, 23, 25, 27, or 29G needle	No treatment	No treatment	2, 4, 6, 8 weeks post insult	X-ray, MRI, histology, IHC, RT-qPCR
10.7150/ijbs.24081	Chen et al., 2018	Berberine suppresses apoptosis and extracellular matrix (ECM) degradation in nucleus pulposus cells and ameliorates disc degeneration in a rodent model	48	8; M	SD	Chloral hydrate	1; C7-C8	Digital palpation + fluoroscopy guided percutaneous puncture with 27G to depth of 4 mm	Immediately after insult	Systemic (intragastric)	0, 4, 8 weeks post insult	MRI, histology, IHC
10.22603/ssrr.2017-0026	Sato et al., 2018	Vascular endothelial growth factor in degenerating intervertebral discs of rat caudal vertebrae	48	250-300 g (~8-10 week); M	SD	Pentobarbital	3; C5-C6 to C7-C8	Open surgery, punctured with 26G needle to a depth of 2 mm 10 times	No treatment	No treatment	1, 7, 14, 28 days	Histoloy, ELISA, IHC
10.3389/fphar.2018.01043	Liu et al, 2018	Urolithin A Inhibits the Catabolic Effect of TNFα on Nucleus Pulposus Cell and Alleviates Intervertebral Disc Degeneration in vivo	30	12; M	SD	Pentobarbital	2; C7-C8, C8-C9	Digital palpation, percutaneous puncture with 21G needle to depth of 5 mm	One day	Systemic (dietary supplement)	4 weeks	X-ray, MRI, histology
10.1038/s41598-017-17289-y	Makino et al., 2017	A selective inhibition of c-Fos/activator protein-1 as a potential therapeutic target for intervertebral disc degeneration and associated pain	32	12; no sex specified	SD	Medetomidine, midazolam, and butorphanol tartrate	2; C6-C7, C7-C8	Digital palpation, percutaneous puncture with 20G needle either to depth of 5 mm, or through entire tail	One day	Systemic (dietary supplement)	4, 8 weeks	X-ray, MRI, histology, IHC, tail-flick latency
10.1016/j.otsr.2017.04.005	Zamora et al., 2017	Effect of Propionibacterium acnes (PA) injection on intervertebral disc degeneration in a rat model: Does it mimic modic changes?	12	400-500 g (~10-14 week); M	SD	Isoflurane	1; C4-C5	Digital palpation, percutaneous puncture with 18G needle to depth of 5 mm	At the time of insult	Intradiscal; 5 μL through 18G	12 Weeks	MRI, histology
10.1007/s00586-016-4898-1	Wang et al., 2017	Repairing the ruptured annular fibrosus by using type I collagen combined with citric acid, EDC and NHS: an in vivo study	48	5-6; M	SD	Pelltobarbitalum natricum	1; C3-C4	Open surgery, puncture with 18G needle	At the time of insult	Focus on annular repair model	4 weeks	X-ray, MRI, histology
10.1016/j.phrs.2017.01.005	Xu et al., 2017	Hydrogen sulfide protects against endoplasmic reticulum stress and mitochondrial injury in nucleus pulposus cells and ameliorates intervertebral disc degeneration	48	12; M	SD	Pentobarbital	1; C7-C8	Digital palpation, percutaneous puncture with 20G needle to depth of 4 mm	Immediately after insult	Systemic (IP delivery)	4 and 8 weeks	MRI, histology
10.1002/jor.23114	Cunha et al., 2017	Joint analysis of IVD herniation and degeneration by rat caudal needle puncture model	14	10; M	W	Isoflurane	3; C5-C6 to C7-C8	Fluoroscopy guided percutaneous puncture with 21 or 25G needle to depth of 5 mm	No treatment	No treatment	2, 6 weeks	X-ray, histology, IHC, biochemical analysis
10.1002/jor.23350	Maidhof et al., 2017	Timing of mesenchymal stem cell delivery impacts the fate and therapeutic potential in intervertebral disc repair	46	350-400 g (~10-12 week); M	SD	N/A	3; C4-C5 to C6-C7	Open surgery, puncture with 27G to depth of 4 mm	3, 14, or 30 days post injury	Open surgery; intradiscal delivery of 5 μL through 33G, cells at 5x10^3/disc	0, 1, 7, or 14 days post treatment	Histology, biomechanic analysis, biochemical analysis
10.18632/oncotarget.14389	Li et al., 2017	Epoxyeicosanoids prevent intervertebral disc degeneration in vitro and in vivo	40	12; M	SD	Diethyl ether	1; C5-C6 to C7-C8	Fluoroscopy guided percutaneous puncture with 21G needle to depth of 5 mm	At the time of insult; 9 more doses given over the next 15 days	Percutaneous puncture; Intradiscal delivery of 2 μL through 31G	4 weeks	X-ray, MRI, histology
10.1007/s12640-016-9676-7	Castania et al., 2017	The Presence of the Neuronal Nitric Oxide Synthase Isoform in the Intervertebral Disk	40	10; M	W	Ketamine-Xylazine	3; C6-C7 to C8-C9	Digital palpation + fluoroscopy guided percutaneous puncture with 21G needle until tactile resistance from contralateral AF was observed	Immediately after insult	Percutaneous puncture; intradiscal delivery of 2 μL through 30G	2 or 21 days	MRI, histology, RT-qPCR, IHC
10.1016/j.yexcr.2017.08.011	Liu et al., 2017	MicroRNA-132 upregulation promotes matrix degradation in intervertebral disc degeneration	60	12; no sex specified	SD	Ketamine-Xylazine	1; C6-C7	Open surgery, puncture with 31G needle to depth of 1.5 mm	At the time of insult	Open surgery; intradiscal delivery through 31G, no volume stated	4 weeks	IHC, RT-qPCR, western blotting
10.1117/12.2255761	Horne et al., 2017	Low intensity pulsed ultrasound (LIPUS) for the treatment of intervertebral disc degeneration	5	12; F	SD	N/A	3; no details	Fluoroscopy guided percutaneous puncture with 20G needle until contralateral AF was reached	30 minutes following insult, and daily for 5 days	Systemic (ultrasound exposure)	5 days	Histology, RT-qPCR
	Hua et al., 2016	The relationship between MRI and histology in a rat model of intervertebral disc degeneration	44	12; M	SD	Pentobarbital	2; C7-C8, C8-C9	Digital palpation guided percutaneous puncture with 20G needle to depth of 5 mm	No treatment	No treatment	4, 8, 12, 24 weeks post-surgery	MRI, histology

10.1007/s10735-015-9651-2	Zhang et al., 2016	Production of CCL20 on nucleus pulposus cells recruits IL-17-producing cells to degenerated IVD tissues in rat models	40	8-10; M	W	Pentobarbital	1; C6-C7	Open surgery, puncture with 20G needle to depth of 5 mm	No treatment for caudal model	No treatment for caudal model	5 weeks	IHC, RT-PCR, western blotting, ELISA
10.3390/ijms17020147	Liao et al., 2016	Cell therapy using bone marrow-derived stem cell overexpressing BMP-7 for degenerative discs in a rat tail disc model	Stage 1: 12; Stage 2: 60	8; M	Lewis (L)	Isoflurane	2; C6-C7, C8-C9	Fluoroscopy guided percutaneous puncture with 18 or 22G needle.	At time of insult, 2 weeks, or 4 weeks after insult	Intradiscal; no needle gauge discussed for treatment delivery; cells delivered at 1x10^4/disc	8 Weeks	X-ray, histology
10.1590/1413-785220162401152960	De Campos et al., 2016	Studies of molecular changes in intervertebral disc degeneration in animal model	12	12; M	W	Ketamine-Xylazine	3; C6-C7 to C8-C9	Fluoroscopy guided percutaneous puncture with 20G needle	No treatment	No treatment	15 or 28 days post puncture	Histology, IHC, RT-PCR
10.1016/j.biomaterials.2015.02.024	Feng et al., 2015	Gene therapy for nucleus pulposus regeneration by heme oxygenase-1 plasmid DNA carried by mixed polyplex micelles with thermo-responsive heterogeneous coronas	32	12; no sex specified	SD	Isoflurane	2; C5-C6, C7-C8	Open surgery puncture with 21G needle to depth of 5 mm	2 weeks	Fluoroscopy guided percutaneous puncture; intradiscal delivery of 2 μL through 31G	6 weeks after initial puncture (4 weeks after treatment)	X-ray, histology, IHC
10.1590/S0102-865020150080000009	Issy et al., 2015	Does a small size needle puncture cause intervertebral disc changes	14	300-350 g (~8-10 week); M	W	Ketamine-Xylazine	3; C7-C8 to C9-C10	Digital palpation + fluoroscopy guided percutaneous puncture with 21G needle	At the time of puncture	Percutaneous intradiscal delivery of 2 μL through 30G	2, 15, 42 days	MRI, Histology
10.1097/BSD.0000000000000141	Inoue et al., 2015	The effect of bone morphogenetic protein-2 injection at different time points on intervertebral disk degeneration in a rat tail model	25	12; M	L	Isoflurane	2; C7-C8, C8-C9	Digital palpation + fluoroscopy guided percutaneous puncture with 18G needle to depth of 5 mm	4, 6, 8 weeks post puncture	Digital palpation percutaneous puncture; intradiscal delivery of 5 μL through 27G	6 weeks post treatment	X-ray, MRI, histology, IHC
10.1016/j.actbio.2015.06.006	Grunert et al., 2015	Riboflavin crosslinked high-density collagen gel for the repair of annular defects in intervertebral discs: An in vivo study	35	10-12; no sex specified	AN	N/A	1; C3-C4	Open surgery, puncture with 18G needle	At time of insult	Focus on annular repair model	5 and 18 weeks	MRI, histology, mechanical testing
10.1371/journal.pone.0113161	Silveira et al., 2014	Protective effects of cannabidiol on lesion-induced intervertebral disc degeneration	19	300-350 g (~8-10 week); M	W	Ketamine-Xylazine	2; C6-C7, C8-C9	Digital palpation guided percutaneous puncture with 21G needle to contralateral AF	At time of insult	Intradiscal; 2 μL through 30G	15 days after the disc puncture	MRI, histology
10.1016/j.spinee.2014.03.050	Ming-Hsiao et al., 2014	Lovastatin prevents discography-associated degeneration and maintains the functional morphology of intervertebral discs	12	12; no sex specified	W	N/A	6; no details	Fluoroscopy guided percutaneous puncture with 27G needle	At time of insult	Intradiscal; 50 μL through 27G	2 or 4 weeks	Histology, IHC, RT-qPCR
10.1016/j.spinee.2013.11.034	Than et al., 2014	Intradiscal injection of simvastatin results in radiologic, histologic, and genetic evidence of disc regeneration in a rat model of degenerative disc disease	272	12; no sex specified	SD	Isoflurane	2; C5-C6, C7-C8	Digital palpation + fluoroscopy guided percutaneous puncture with 21G needle to depth of 5 mm	6 weeks post insult	Percutaneous intradiscal delivery of 2 μL through 31G	2, 4, 8, 12, and 24 weeks post treatment	MRI, histology, RT-PCR
10.1097/BRS.0000000000000194	Grunert et al., 2014	Assessment of intervertebral disc degeneration based on quantitative magnetic resonance imaging analysis: An in vivo study	24	190-250 g (~8-10 week); M	AN	Isoflurane	1; C3-C4	Independent sets testing open surgery and percutaneous punctures, both with 18G needle	At time of insult	Focus on annular repair model	1 and 3 months	MRI, histology
10.1097/BRS.0000000000000103	Grunert et al., 2014	Annular repair using high-density collagen gel: A rat-tail in vivo model	42	10-12; M	AN	N/A	1; C3-C4	Open surgery, puncture with 18G needle	At time of insult	Focus on annular repair model	5 weeks	MRI, X-ray, histology
10.1177/0885328213515034	Yan et al., 2014	Effects of releasing recombinant human growth and differentiation factor-5 from poly(lactic-co-glycolic acid) microspheres for repair of the rat degenerated intervertebral disc	N/A	12; M	SD	Chloral hydrate	3; C4-C5, C5-C6, C7-C8	Open surgery, puncture with 21G needle	4 weeks post insult	Intradiscal; unknown volume, through 31G	8 weeks post treatment	X-ray, MRI, histology, biochemical analysis, RT-qPCR
10.1016/j.actbio.2013.08.019	Liang et al., 2013	Dual release of dexamethasone and TGF-β3 from polymeric microspheres for stem cell matrix accumulation in a rat disc degeneration model	96	12; M	SD	Pentobarbital	2; C7-C8, C8-C9	Digital palpation percutaneous puncture with 20G needle to depth of 5 mm	2 weeks post injury	Intradiscal; 2 μL through 31G, cells at 1x10^6 cells/mL	4, 8, 16, 24 weeks post treatment	X-ray, MRI, histology, IHC, biochemical analysis, RT-qPCR

10.3892/mmr.2013.1450	Zou et al., 2013	Efficacy of intradiscal hepatocyte growth factor injection for the treatment of intervertebral disc degeneration	30	200-250 g (~6-8 week); M	SD	Ketamine-Xylazine	2; C5-C6, C7-C8	Fluoroscopy guided percutaneous puncture with 21G needle	4 weeks post insult	Intradiscal delivery; 2 µL through 31G	2 or 4 weeks post treatment	MRI, histology, IHC
10.1186/ar4224	Rastogi et al., 2013	MMP-2 mediates local degradation and remodeling of collagen by annulus fibrosus cells of the intervertebral disc	N/A	26-32; no sex specified	SD	Isoflurane	1; C6-C7	Open surgery with fluoroscopy to confirm appropriate depth; puncture with either 26G, 22G, or 18G	No in vivo treatment	No in vivo treatment	2 weeks	Histology, IHC
10.1590/1414-431X20122429	Issy et al., 2013	Experimental model of intervertebral disc degeneration by needle puncture in Wistar rats	16	300-350 g (~8-10 week); M	W	Ketamine-Xylazine	2; C6-C7, C8-C9	Digital palpation + fluoroscopy guided puncture with 20G needle, until resistance from contralateral AF was observed	No treatment	No treatment	7 or 30 days post puncture	X-ray, MRI, histology
10.1016/j.spinee.2013.01.040	Tsai et al., 2013	Increased periostin gene expression in degenerative intervertebral disc cells	8	8; M	L	N/A	2; C6-C7, C8-C9	Digital palpation + fluoroscopy guided puncture with 18G needle	No treatment	No treatment	8 weeks post puncture	Histology, IHC
10.1590/S1413-78522013000300003	Oliveira et al., 2013	Extracellular matrix remodeling in experimental intervertebral disc degeneration	3	12; M	W	Ketamine-Xylazine	2; C6-C7, C8-C9	Digital palpation, percutaneous puncture with 20G needle	No treatment	No treatment	0, 15, 30 days	IHC
10.3171/2011.5.SPINE10811	Zhang et al., 2011	Time course investigation of intervertebral disc degeneration produced by needle-stab injury of the rat caudal spine: Laboratory investigation	32	12; M	SD	Isoflurane	2; C5-C6, C7-C8	Fluoroscopy guided percutaneous puncture with 21G needle to depth of 5 mm	No treatment	No treatment	3, 10, 17, 42 days	MRI, histology, RT-qPCR, biochemical analysis
10.1016/j.spinee.2010.08.013	Keorochana et al, 2010	The effect of needle size inducing degeneration in the rat caudal disc: Evaluation using radiograph, magnetic resonance imaging, histology, and immunohistochemistry	36	12-14; M	L	Isoflurane	2; C6-C7, C8-C9	Digital palpation + fluoroscopy guided percutaneous puncture with 18, 20, or 22G needle to depth of 5 mm	No treatment	No treatment	2, 4, 6, 8 weeks	X-ray, MRI, histology, IHC
10.1186/ar2861	Zhang et al., 2009	Intradiscal injection of simvastatin retards progression of intervertebral disc degeneration induced by stab injury	30	12; no sex specified	SD	Isoflurane	2; C5-C6, C7-C8	Open surgery using fluoroscopy to guide puncture depth; puncture using 21G needle to depth of 5 mm	4 weeks post insult	Intradiscal delivery of 2 µL through 31G	2 weeks post treatment	MRI, histology, biochemical analysis, RT-qPCR
10.3171/2009.4.SPINE08744	Zhang et al., 2009	The effects of punctured nucleus pulposus on lumbar radicular pain in rats: A behavioral and immunohistochemical study - Laboratory investigation	72	200-250 g (~6-8 week); M	SD	Pentobarbital	2; C4-C5, C8-C9	Open surgery, puncture with 21G needle to depth of 3 mm	No treatment on caudal model	No treatment on caudal model	2 weeks	MRI, histology
10.3171/2009.2.SPINE08925	Zhang et al., 2009	Developing consistently reproducible intervertebral disc degeneration at rat caudal spine by using needle puncture - Laboratory investigation	9	12; M	SD	Isoflurane	2; C5-C6, C7-C8	Open surgery + fluoroscopy guided puncture with 18G or 21G to depth of 5 mm	No treatment	No treatment	3 months	MRI, histology, IHC
10.1097/BRS.0b013e31819c09c4	Hsieh et al., 2009	Degenerative anular changes induced by puncture are associated with insufficiency of disc biomechanical function	36	32-36; no sex specified	SD	Isoflurane	1; C6-C7	Fluoroscopy guided percutaneous puncture with 18, 22, or 26G needle to depth of 2 mm	No treatment	No treatment	1, 2, 4 weeks	Histology; mechanical testing done in parallel in organ culture model
10.1097/BRS.0b013e31817c64a9	Han et al., 2008	A simple disc degeneration model induced by percutaneous needle puncture in the rat tail	163	12; M	SD	Chloral hydrate	2; C7-C8, C8-C9	Digital palpation + fluoroscopy guided percutaneous puncture with 20G needle either to depth of 5 mm or through contralateral aspect of tail	No treatment	No treatment	1, 2, 4 weeks	X-ray, histology, biochemical analysis
10.1097/BRS.0b013e31815b9850	Ulrich et al. 2007	Repeated disc injury causes persistent inflammation	48	12; sex not specified	SD	Ketamine-Xylazine	3; C5-C6 to C7-C8	Open surgery and disc stab with number 11 blade, then fluoroscopy guided percutaneous puncture with 23G needle to depth of 1.5 mm (either single stab or triple stab)	No treatment	No treatment	Single-stab discs were examined 4, 7, 14, 28, and 56 days. Triple-stab discs were examined 9, 14, 28, and 56 days	Histology, ELISA, IHC
10.1097/01.brs.0000251013.07656.45	Rousseau et al., 2007	Stab Incision for Inducing Intervertebral Disc Degeneration in the Rat	24	12; sex not specified	SD	Ketamine-Xylazine	3; C5-C6 to C7-C8	Open surgery, stab with number 11 blade to depth of 1.5 mm	No treatment	No treatment	4, 7, 14, 28 days	Histology, ELISA, ex vivo mechanical testing
10.1080/15476286.2021.1898176	Wang et al. 2021	MicroRNA-140-3p alleviates intervertebral disc degeneration via KLF5/N-cadherin/MDM2/Slug axis	64	12; M	SD	Chloral hydrate	1; C7-C8	Fluoroscopy guided percutaneous puncture with 21G needle	Immediately	Intradiscal delivery of treatment; no volume or needle gauge reported	4, 7, 14, 28 days post puncture	X-ray, histology, RT-qPCR

10.3389/fcell.2021.687024	Liu et al. 2021	Fexofenadine protects against intervertebral disc degeneration through TNF signaling	18	8-12; M	SD	N/A	1; C8-C9	Digital palpation, percutaneous puncture with 20G needle to depth of 5 mm	Every other day	Systemic (intraperitoneal) treatment delivery	7 days	Histology, RT-PCR, western blotting, ELISA, IHC
10.1016/j.lfs.2021.119874	Wang et al. 2021	17Beta-estradiol alleviates intervertebral disc degeneration by inhibiing NF-kB signal pathway	18	12; F	SD	N/A	1; C6-C7	Digital palpation guided percutaneous puncture with 21G needle to depth of 3 mm	3 days post insult	Systemic (subcutaneous treatment delivery)	8 weeks	X-ray, MRI, histology, IHC
10.1155/2021/6632786	Yu et al. 2021	Mangiferin alleviates mitochondrial ROS in nucleus pulposus cells and protects against intervertebral disc degeneration via suppression o fNF-kB signaling pathway	15	12; no sex specified	SD	Pentobarbital	2; C8-C9, C9-C10	Digital palpation + fluoroscopy guided percutaneous puncture with 20G needle to depth of 5 mm	3 days post insult	Intradiscal; 2 µL through 33G	7 days after initial puncture, 4 days after treatment	X-ray, MRI, histology, IHC
10.3390/ijms222111355	Kim et al. 2021	Activation of hypoxia-inducible factor-1alpha signaling pathway has the protective effect of intervertebral disc degeneration	10	8; M	SD	Isoflurane	4; C4-C5 to C7-C8	Fluoroscopy guided percutaneous puncture with 21G needle	At time of insult	Intradiscal delivery of unknown volume through 21G needle	8 weeks	X-ray, MRI, histology, IHC
10.1007/s10495-022-01707-2	Wu et al. 2022	SKI knockdown suppresses apoptosis and extracellular matrix degradation of nucleus pulposus cells via inhibition of the Wnt/β-catenin pathway and ameliorates disc degeneration	40	200-230 g (~6-8 week); M	SD	Pentobarbital	1; C7-C8	Digital palpation guided percutaneous puncture with 21G needle	At time of insult	Intradiscal delivery of 2 µL through 21G	8 weeks	Histology, MRI, RT-qPCR, western blotting, IHC
10.1038/s12276-022-00729-9	Zhang et al. 2022	Cytosolic escape of mitochondrial DNA triggers cGAS-STING-NLRP3 axis-dependent nucleus pulposus cell pyroptosis	7	12; sex not specified	SD	Pentobarbital	2; C8-C9, C9-C10	Digital palpation guided percutaneous puncture with 29G needle to depth of 5 mm	At time of insult and repeated weekly for 1 month	Intradiscal percutaneous delivery of 2 µL through 29G	1 month	X-ray, MRI, histology, IHC
10.1186/s10020-021-00351-x	Cheng et al. 2021	CB2-mediated attenuation of nucleus pulposus degeneration via the amelioration of inflammation and oxidative stress in vivo and in vitro	40	400 g (~10-14 week); M	SD	Chloral hydrate	1; C7-C8	Fluoroscopy guided percutaneous puncture with 21G needle	Weekly	Open surgery, intradiscal delivery of 2 µL, no needle gauge stated for treatment delivery	1, 2, 3, 4 weeks	X-ray, MRI, histology, IHC
10.1038/s41598-021-94173-w	Matta et al. 2021	A comparative study of mesenchymal stem cell transplantation and NTG-101 molecular therapy to treat degenerative disc disease	30	12; F	W	Isoflurane	5; no details	Fluoroscopy guided percutaneous puncture with 26G needle through contralateral AF	10 weeks post injury	Percutaneous intradiscal delivery of 8 µL through 32G; cells at 150,000 cells/disc	10 weeks post treatment	Histology, IHC, western blotting, RT-qPCR
